# Chemistry: General, Medical, and Pharmaceutical

**Published:** 1890-01

**Authors:** 


**Chemistry: General, Medical, and Pharmaceutical,** including the Chemistry of the U. S. Pharmacopeia. By JOHN ATTFIELD, F. R. S. Twelfth edition. Philadelphia: Lea Brothers & Co. Pp. 770. 1889.

A work which has passed through twelve consecutive editions needs no comment, no word of praise, from the reviewer's pen. This edition contains such alterations and additions as are necessary to keep abreast with the latest observations and latest applications of chemistry in pharmacy. The work now includes the whole of the chemistry of the United States Pharmacopeia, and nearly all the chemistry of the British and Indian Pharmacopeias.

The section on Organic Chemistry is classified according to the system now adopted by most authorities.

The typography is up to the standard of previous editions.

